# Targeting a highly repeated germline DNA sequence for improved real-time PCR-based detection of *Ascaris* infection in human stool

**DOI:** 10.1371/journal.pntd.0007593

**Published:** 2019-07-22

**Authors:** Nils Pilotte, Jacqueline R. M. A. Maasch, Alice V. Easton, Eric Dahlstrom, Thomas B. Nutman, Steven A. Williams

**Affiliations:** 1 Department of Biological Sciences, Smith College, Northampton, MA, United States of America; 2 Molecular and Cellular Biology Program, University of Massachusetts, Amherst, MA, United States of America; 3 Helminth Immunology Section, Laboratory of Parasitic Diseases, National Institute of Allergy and Infectious Disease, National Institutes of Health, Bethesda, MD, United States of America; 4 Genomics Unit, Research Technologies Section, Rocky Mountain Laboratories, National Institute of Allergy and Infectious Diseases, National Institutes of Health, Hamilton, MT, United States of America; University of Cambridge, UNITED KINGDOM

## Abstract

**Background:**

With the expansion of soil transmitted helminth (STH) intervention efforts and the corresponding decline in infection prevalence, there is an increased need for sensitive and specific STH diagnostic assays. Previously, through next generation sequencing (NGS)-based identification and targeting of non-coding, high copy-number repetitive DNA sequences, we described the development of a panel of improved quantitative real-time PCR (qPCR)-based assays for the detection of *Necator americanus*, *Ancylostoma duodenale*, *Ancylostoma ceylanicum*, *Trichuris trichiura*, and *Strongyloides stercoralis*. However, due to the phenomenon of chromosome diminution, a similar assay based on high copy-number repetitive DNA was not developed for the detection of *Ascaris lumbricoides*. Recently, the publication of a reference-level germline genome sequence for *A*. *lumbricoides* has facilitated our development of an improved assay for this human pathogen of vast global importance.

**Methodology/Principal findings:**

Repurposing raw DNA sequence reads from a previously published Illumina-generated, NGS-based *A*. *lumbricoides* germline genome sequencing project, we performed a cluster-based repeat analysis utilizing RepeatExplorer2 software. This analysis identified the most prevalent repetitive DNA element of the *A*. *lumbricoides* germline genome (AGR, *Ascaris* germline repeat), which was then used to develop an improved qPCR assay. During experimental validation, this assay demonstrated a fold increase in sensitivity of ~3,100, as determined by relative Cq values, when compared with an assay utilizing a previously published, frequently employed, ribosomal internal transcribed spacer (ITS) DNA target. A comparative analysis of 2,784 field-collected samples was then performed, successfully verifying this improved sensitivity.

**Conclusions/Significance:**

Through analysis of the germline genome sequence of *A*. *lumbricoides*, a vastly improved qPCR assay has been developed. This assay, utilizing a high copy-number repeat target found in eggs and embryos (the AGR repeat), will improve prevalence estimates that are fundamental to the programmatic decision-making process, while simultaneously strengthening mathematical models used to examine STH infection rates. Furthermore, through the identification of an optimal target for PCR, future assay development efforts will also benefit, as the identity of the optimized repeat DNA target is likely to remain unchanged despite continued improvement in PCR-based diagnostic technologies.

## Author summary

With an at-risk population in the billions, *Ascaris lumbricoides* is a pathogen of great global importance. In recent years, efforts to control the spread of this parasitic helminth have expanded, resulting in declining infection rates and worm burdens in some regions. While immeasurably important for global health, these declines have also served to expose the shortcomings of traditional diagnostic methods, as low-levels of pathogen generate a need for more sensitive tools, and microscopy-based techniques are proving ill-suited to the task at hand. Thankfully, improved sensitivity can be achieved through the careful selection of optimal repetitive DNA targets for PCR. However, previous attempts to identify such targets in *A*. *lumbricoides* were unsuccessful, largely due to chromosome diminution, an unusual phenomenon occurring in the Ascaridida, whereby large portions of the germline genome are reproducibly eliminated during early development, resulting in their absence in larvae or adult worms. As the stool-based molecular diagnosis of *A*. *lumbricoides* infection is primarily dependent upon the identification of egg-derived DNA, utilizing genomic DNA from adult worms for molecular target selection eliminates germline candidates and results in suboptimal target sequence choices. Recently, the publication of a pre-diminution germline genome of *A*. *lumbricoides* has provided us with an opportunity to re-evaluate target selection, facilitating the development of a novel quantitative real-time PCR assay with greatly improved sensitivity (~3100-fold as determined by relative Cq value) over previously developed assays that were based on ribosomal repeat DNA sequences with lower copy numbers.

## Introduction

Believed responsible for more than 800 million global infections, *Ascaris lumbricoides* is the most prevalent of the human-infecting soil transmitted helminths (STH) [1–2]. As recently as 2017, infections with this parasite were believed to result in approximaely 861,000 disability adjusted life years [3], generating nearly 45% of the global years lived with disability attributable to the overall burden of STH infections [3]. Due to an improved understanding of the scope of this disease burden, there is now an increased recognition of the global health impact of *A*. *lumbricoides* and the other STH infections. Such awareness has resulted in the expansion of infection and risk mapping efforts [4–9] and operational research studies intended to improve, expand, and more fully understand the impacts associated with interventions [10–15]. Similarly, due to exponential improvements in approaches to mathematical modelling, the roles played by these valuable tools for shaping and informing the STH programmatic decision making process continue to increase [5–6, 16–18]. Fundamentally, such operational research efforts and modelling strategies rely heavily upon the availability of accurate data. Such reliance is particularly critical following interventions that have resulted in declining prevalence, drawing greater attention to the ramifications of employing insensitive diagnostic methods such as Kato-Katz [19]. Therefore, sensitive and specific diagnostic tools facilitating the collection of accurate data are increasingly critical for the proper interpretation of findings and the veracity of resulting conclusions.

Previously, we described a pipeline for the identification of high-copy number repetitive DNA elements for use as semi-quantitative real-time PCR (qPCR) targets for the detection of various STH species [20–21]. These targets, identified utilizing next-generation sequencing (NGS)-based analysis tools, have facilitated improved sensitivity and specificity of detection, leading to their adoption in various diagnostic efforts and operational research (OR) studies such as the DeWorm3 cluster randomized trials [11, 14, 22]. Despite the availability of such tools, to date, the qPCR-based detection of *A*. *lumbricoides* has depended upon less optimal targets, such as ribosomal internal transcribed spacer (ITS) sequences [20, 23–24]. This shortcoming is rooted in the unique process of chromosome diminution, whereby some species, including certain members of the order Ascaridida, undergo programmed elimination of select and reproducible regions of their gDNA during development [25–26]. In the case of *A*. *lumbricoides*, diminution occurs between the third and seventh embryonic divisions [27], and an estimated 13% of the haploid germline genome is eliminated by this process [25], including the most abundant of the genome’s tandemly repeated sequences [26]. Such elimination of highly repetitive, non-coding sequences during embryonic development renders ribosomal repeats the highest copy number gDNA sequences remaining in the genomes of larval and adult *Ascaris* worms. As pure gDNA is more easily obtained from adult worms, initial analyses using our pipeline utilized adult DNA extracts, and therefore failed to identify repeats present at higher copy number than the ribosomal ITS sequences [20]. However, STH diagnosis is dependent upon the detection of DNA from eggs/early embryos extracted from the stool of infected individuals. Thus, identifying an optimal qPCR target requires examination of egg-derived DNA, possessing pre-diminution gDNA sequences.

Acknowledging this shortcoming in the currently available PCR diagnostic toolkit, we now describe the development of an *Ascaris* germline assay utilizing a highly repetitive DNA element whose copy number is reduced by an estimated 99% in the post-diminution genome of larval and adult worms [26]. This 120 bp target, hereafter referred to as the *Ascaris* germline repeat (AGR), was previously estimated to constitute approximately 8.9% of the *Ascaris* germline genome [26], and further analysis of germline sequence reads utilizing RepeatExplorer2, a Galaxy-based computational tool [28] supports the prediction that this tandem repeat represents the most abundant germline gDNA sequence. The incorporation of a new PCR-based assay utilizing this improved target into our previously described STH diagnostic pipeline [20–21], represents a significant diagnostic improvement with the capacity to aid future programmatic efforts.

## Materials and methods

### Ethics statement

The use of human samples in this study was approved by the reviewing body at the International Centre for Diarrhoeal Disease Research, Bangladesh (protocol # PR-14105) and by the University of California at Berkeley Committee for Protection of Human Subjects (protocol # 2014-08-6658).

### Repetitive DNA sequence analysis

**Fig 1:**
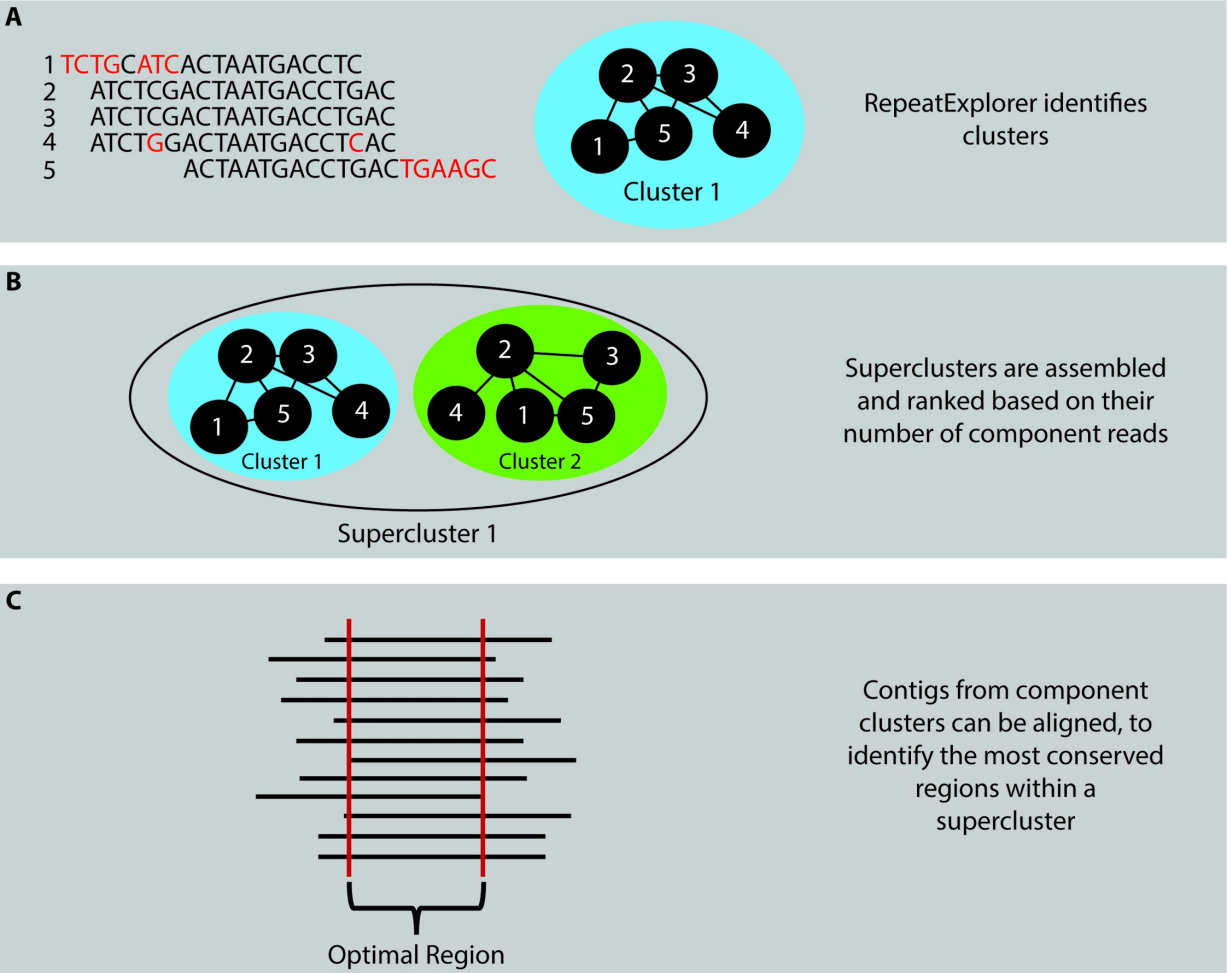
Identification of an optimal PCR target using RepeatExplorer2. (A) By comparing the individual sequence of each read to the sequence of every other read within the dataset of interest, RepeatExplorer builds “clusters” from reads meeting the cut-off criteria of having 90% or greater sequence identity over 55% or more of the read lengths. (B) Further comparison then identifies superclusters, comprised of clusters reaching a threshold level for paired-end read mates shared between clusters. (C) By aligning contigs/reads comprising component clusters within a supercluster, it becomes possible to identify regions of DNA sequence within each supercluster that have the greatest coverage. These highly repeated DNA regions are selected as targets for qPCR reaction primer and probe design.

Three independently prepared, paired-end DNA libraries of raw Illumina sequencing reads, previously utilized for the assembly of a reference-quality *A*. *lumbricoides* germline genome [26], were repurposed for use in this study (Sequence Read Archive [SRA] BioProject number PRJNA511996). Prior to SRA upload, all reads were trimmed to a uniform length of 93 bp and a full description of sequencing and filtering methodologies has been described elsewhere [26]. Utilizing a randomly selected subset of 500,000 reads, each paired-end library was analyzed using RepeatExplorer2, a Galaxy-based analysis tool for the identification of repetitive DNA elements [28]. These analyses were used to identify the highest copy number genomic DNA sequences, which were selected as PCR targets for further analysis (Fig 1). All RepeatExplorer analyses were performed using default settings, without advanced options, and with the “Select Queue” set to “Basic and Fast”.

### Assay design

Utilizing default parameters for PrimerQuest Tool software (Integrated DNA Technologies, Coralville, IA), a candidate primer-probe pairing was designed that targeted our identified DNA repeat sequence. Primer-BLAST, available from the National Center for Biotechnology Information (NCBI) website (www.ncbi.nlm.nih.gov), was employed to determine whether or not our candidate primers matched off-target template sequences found within the RefSeq Representative Genome Database, and NCBI’s nucleotide collection database. Following analysis, primers and probe were synthesized by Integrated DNA Technologies. Probe chemistry included labeling with a 6-FAM fluorophore at the 5’ end, and double quenching with ZEN (internal) and 3IABkFQ (3’ end) chemistries (Table 1).

**Table 1:** Primer and probe sequences for the AGR assay.

Forward Primer	5’–CTTGTACCACGATAAAGGGCAT—3’
Reverse Primer	5’- TCCCTTCCAATTGATCATCGAATAA—3’
Probe	5’—/56-FAM/TCTGTGCAT/ZEN/TATTGCTGCAATTGGGA/3IABkFQ/ - 3’

### Assay validation and optimization

Assay validation and optimization experiments were performed as previously described [20–21]. Briefly, utilizing 200 pg of pure *A*. *lumbricoides* gDNA isolated from an adult female worm as template, optimal primer concentrations were determined by titrating forward and reverse primers in independent 7 μL reactions containing 3.5 μL of TaqPath ProAmp Master Mix (ThermoFisher Scientific, Waltham, MA). Employing doubling dilutions, primers were tested at concentrations ranging from 1000 nM to 62.5 nM, with forward and reverse primer concentrations tested in all possible dilution combinations. Optimal AGR primer concentrations were then utilized in reactions intended to verify assay specificity, whereby 2 ng of purified genomic DNA isolated from adult *Necator americanus*, adult *Ancylostoma duodenale*, adult *Ancylostoma ceylanicum*, adult *Trichuris trichiura*, *Strongyloides stercoralis* L1 larvae, adult *Schistosoma mansoni*, adult *Anisakis typica*, adult *Baylisascaris procyonis*, and adult *Parascaris univalens*, were used as template in separate reactions. Additionally, testing against human DNA, gDNA from *Candida albicans* (strain L26) (BEI Resources, Manassas, VA), DNA from the common gut bacteria *Escherichia coli*, and gDNA from a “mock” microbial community (v5.2H) (BEI Resources) also occurred. As a final validation, a panel of 20 infection-naïve, commercially available human stool samples were obtained for testing (BioIVT, Westbury, NY). DNA extraction was performed as previously described [29] and each extract was then tested for the presence of *Ascaris* signal.

### Generation of a plasmid control containing the assay target sequence

Utilizing our AGR qPCR assay primers, pure *A*. *lumbricoides* gDNA was amplified by conventional PCR. Reactions in 25 μL volumes, containing 12.5 μL of Q5 Hot Start High-Fidelity 2X Master Mix (New England Biolabs, Ipswich, MA) and 500 nM concentrations of each primer were amplified with an initial 30 second incubation at 98°C; followed by 35 cycles of 98°C for 10 seconds, 63°C for 30 seconds, and 72°C for 30 seconds; and a final 2 minute extension step at 72°C. Following cycling, PCR products were cloned into the pCR-Blunt II-TOPO vector (ThermoFisher Scientific) in accordance with the manufacturer’s suggested protocol, and NEB Express Competent *E*. *coli* (New England Biolabs) were transformed with 3 μL of the ligated plasmid. Transformed competent cells were then plated on LB-kanamycin plates, and grown at 37°C overnight. Colonies were picked, and colony PCR was performed in 25 μL reactions containing 12.5 μL of One Taq 2x Master Mix (New England Biolabs), with 500 nM M13 forward and reverse primers. Cycling began with an initial 30 second denaturation at 94°C, followed by 35 reaction cycles of 94°C for 15 seconds, 44°C for 30 seconds, and 68°C for 90 seconds; and a final extension for 5 minutes at 68°C. Reaction products were sequenced, and a plasmid clone containing a single copy of the correct AGR repeat element was selected for use as a positive control in all future experiments.

### Determination of assay efficiency

In order to determine assay efficiency, a panel of 10-fold plasmid serial dilutions was generated. Dilutions ranged from 100 pg/μL to 100 ag/μL. Because the control plasmid is 3,595 bp in size, and the average mass of a single nucleotide base pair is estimated to be 650 Da, 100 ag of plasmid was estimated to correspond to approximately 50 copies of the plasmid. Utilizing this information, approximate copy numbers were calculated for each concentration within the serial dilution series. Optimized reaction conditions were then employed to perform 11 or 12 reaction replicates for each dilution. Mean Cq values were calculated for reactions performed on each concentration of template, and a reaction efficiency was calculated.

### Determination of assay detection limits

To determine assay detection limits, a panel of banked DNA extracts, previously isolated (as described elsewhere [29]) from 50 mg naïve stool samples that had been spiked with known numbers of *A*. *lumbricoides* eggs, was tested for the presence of detectable levels of *A*. *lumbricoides* target DNA. These samples were prepared and extracted as part of an ongoing, unrelated study in which the identification and isolation of eggs utilized for spiking was performed using the McMaster egg counting technique as previously described [30]. Eggs were carefully removed from their parent samples under the microscope, briefly rinsed in nuclease-free water, and then added to the naïve stool. Following the addition of eggs, DNA was extracted from spiked aliquots as previously described [29]. All testing occurred in duplicate, and was performed using the experimentally-determined optimal AGR assay conditions. To facilitate inter-assay comparison, samples were similarly tested using a previously described assay that targets a ribosomal ITS2 sequence [20]. In total, 19 samples were tested. Four samples were spiked with 40 eggs, four with 10 eggs, four with 5 eggs, and four with 2 eggs. An additional three samples containing DNA from a single egg completed the panel.

### Assay validation utilizing field-collected samples

#### Collection of samples and assessment of quality

A panel of 2,784 previously isolated (as described elsewhere [29]) human stool DNA extracts were utilized in this study. All samples were collected in Bangladesh as part of the WASH Benefits Bangladesh trial [15]. At the time of processing, extraction quality was assessed, as previously described [29], through the procedural inclusion of an internal amplification control (IAC) plasmid. Following the extraction of all DNA samples, the recovery of IAC plasmid, spiked into each sample during the extraction procedure, was assessed using qPCR, and a mean Cq value and standard deviation (SD) was calculated for all samples. To ensure extraction quality and data comparability, all samples which produced a Cq value > 3SD from the mean underwent re-extraction and re-testing.

#### qPCR testing

All samples and standards were tested in duplicate, utilizing the optimized version of the newly described AGR assay. After assaying all samples, results for each sample were compared with results obtained using the previously described ITS2-targeting assay referenced above [20]. While testing with both assays occurred sequentially, rather than simultaneously, samples were properly stored at -20°C until all testing was completed. On all experimental plates, plasmid controls were run at concentrations of 10 pg/μL, 100 fg/μL, and 1 fg/μL. Following the completion of all testing, standard deviations were calculated for the mean Cq values for each control concentration. For a given experimental plate, in the event that the mean Cq value for any control dilution was > 2SD from the mean, the entire plate was retested. In the event that a given sample produced one positive and one negative result, the sample was re-tested in duplicate. For such samples, a positive result was ascribed to the sample if it again produced one or more positive results upon re-testing. Samples which produced two negative results upon retesting were considered to be negative. All criteria for test validity and sample positivity/negativity determination were identical to those used for testing with the ITS2-targeting assay.

#### Statistical analysis

An agreement table was generated to assess assay concordance/discordance. Samples were scored as “true positives” when they were found to be positive by either experimental assay. Assay sensitivities were calculated by dividing the number of positives as determined by a given assay, by the total number of “true positives”.

## Results

### Target sequence identification and assay design

RepeatExplorer2 analysis software was employed to identify genomic DNA elements of putatively greatest copy number from sequence data derived from three, previously prepared, paired-end libraries of egg-derived *A*. *lumbricoides* DNA [26]. For each library, the RepeatExplorer-generated cluster containing the largest number of DNA sequence reads contained a 120 bp satellite element, previously identified as the most numerous within the *A*. *lumbricoides* germline genome [26]. Similarly, for each analyzed library, a supercluster comprised of multiple clusters each mapping to this repeat was predicted to represent between 7.9% and 16.3% of the germline genome. While it is important to note that such superclusters contain additional sequence fragments (flanking regions, etc.), when considered as rough representations of a sequence’s genome percentage, these estimates are consistent with the 8.9% prediction made by Wang, et al [26]. These results strongly suggest that this repetitive sequence is the most prevalent repeat DNA element within the germline genome of *A*. *lumbricoides*. Utilizing this 120 bp AGR sequence, PrimerQuest Tool software was employed to design a candidate primer-probe set and Primer-BLAST analysis of this set returned only *Ascaris*-derived product predictions, minimizing the likelihood of experimental off-target PCR amplification.

### Assay validation and optimization

As previously described, a titration of doubling dilutions of primer candidates was employed to determine optimal primer concentrations [20]. As determined by mean Cq value, optimal concentrations were determined to be 125 nM for the forward primer and 500 nM for the reverse primer. Utilizing these primer concentrations, assay specificity was verified: 2 ng of template failed to produce off-target amplification for any of the species or samples tested. Similarly, testing of all DNA extracts from the infection-naïve stool panel failed to produce *Ascaris* signal, indicating that cross-reactivity with common elements of the gut flora is unlikely to occur.

### Determination of assay efficiency

**Fig 2:**
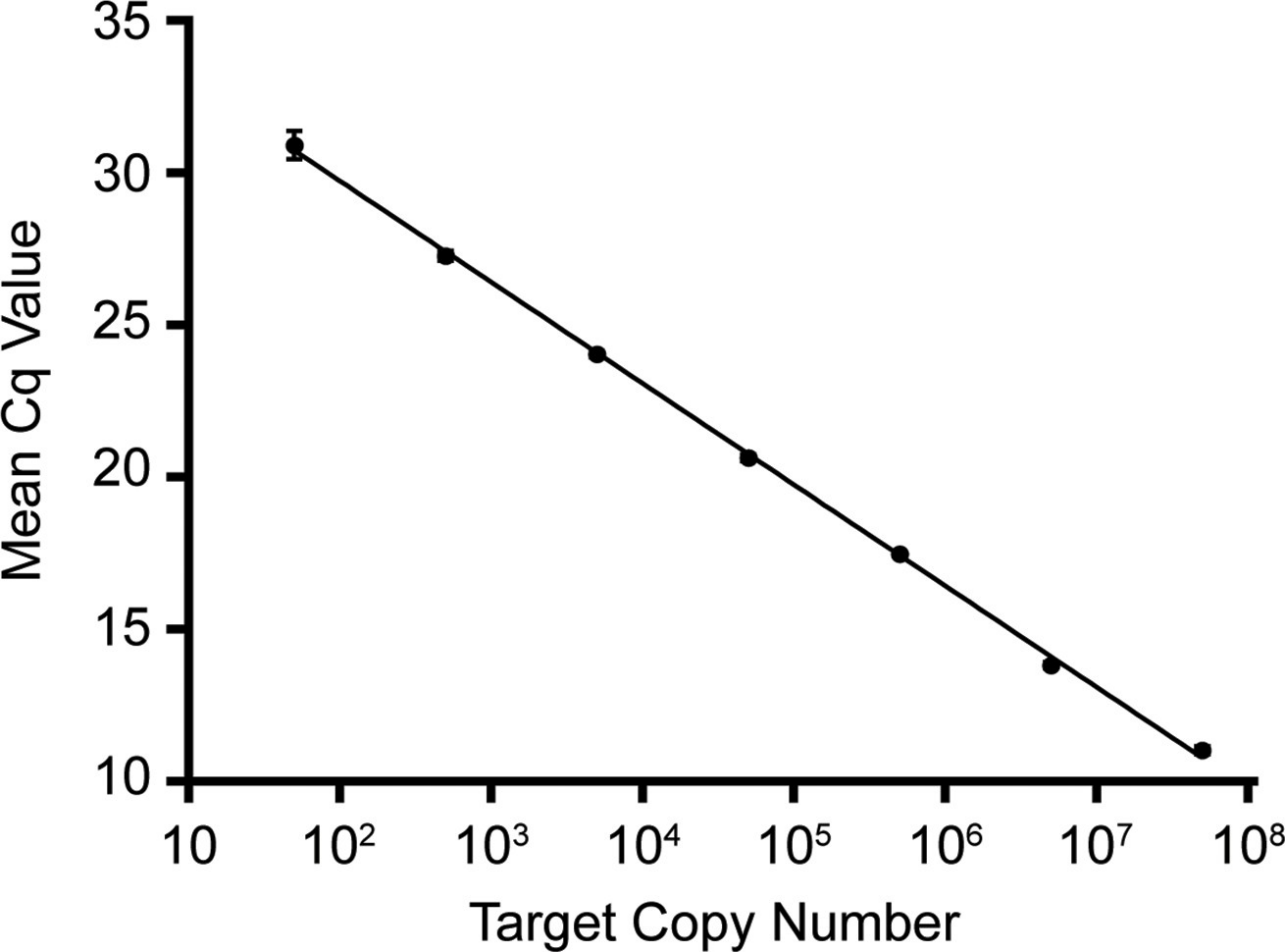
Calculation of assay efficiency. To determine assay efficiency, 10-fold serial dilutions of control plasmid were prepared. All dilutions, ranging in concentration from 100 pg/μL to 100 ag/μL, were analyzed in either 11 or 12 replicate reactions. Mean Cq values and standard deviations were then calculated for each concentration of plasmid template, a slope was plotted, and reaction efficiency and amplification factor were determined.

By testing a titration of our generated control plasmid (S1 Fig), assay efficiency was determined. Utilizing plasmid size to determine target copy number per titration, a standard curve was generated by plotting target copy # vs. mean Cq value (Fig 2). The slope of this curve was determined to be -3.3216, with a reaction efficiency of 100.1% and an amplification factor of 2.00.

### Determination of assay detection limits

Utilizing DNA extracts obtained from naïve stool samples spiked with known numbers of *A*. *lumbricoides* eggs, assay detection limits were determined. Results using the new AGR assay indicated that target detection was possible from all stool samples spiked with all tested concentrations of eggs ranging from 40 eggs to a single egg (Table 2). In contrast, results obtained when testing with the ITS-targeting assay failed to allow for consistent detection at both 1 and 2 egg concentrations (Table 2).

**Table 2:** Evaluation of limits of detection utilizing DNA extracts from spiked stool samples.

Number of Samples	Number of Eggs per Sample (EPG)	Mean Cq Value [Range]^a^ AGR Assay	Mean Cq Value [Range]^a^ ITS Assay
4	40 (800)	15.61 [14.69–16.77]	25.40 [24.69–26.65]
4	10 (200)	15.99 [14.43–17.36]	25.60 [24.11–26.78]
4	5 (100)	20.16 [18.52–21.85]	30.56 [28.36–32.81]
4	2 (40)	25.26 [20.85–29.65]	32.76 [31.33–33.85]^b^
3	1 (20)	24.21 [19.12–32.44]^d^	30.21 [28.46–32.87]^c^
2	No Template Control	0.00 [0.00–0.00]	0.00 [0.00–0.00]

^**a**^ Each spiked sample was run in duplicate, resulting in a mean for each sample. Mean Cq values for a given egg concentration were calculated as the mean value of all component sample means. The reported “Range” is the lowest individual Cq value and the highest individual Cq value for a given egg concentration. EPG, eggs per gram.

^b^ Only two samples resulted in amplification of *Ascaris* target, with one of these samples amplifying in only 1 of 2 replicate reactions.

^c^ Only two samples resulted in amplification of *Ascaris* target.

^d^ One sample amplified in only 1 of 2 replicate reactions.

### Assay validation utilizing field-collected samples

**Fig 3:**
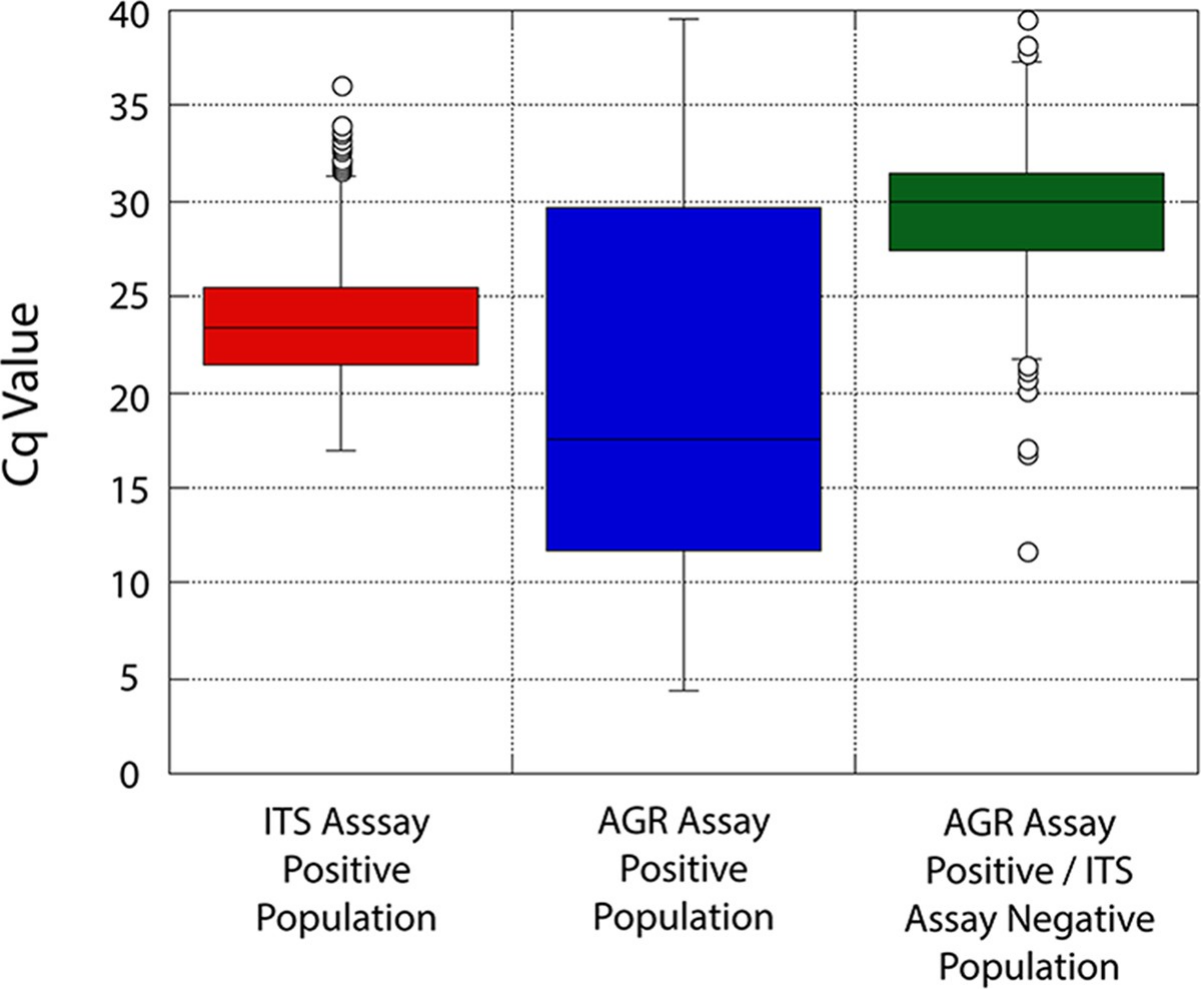
Boxplots of positive sample populations from field-collected samples. Plots represent the total population of ITS-assay-positive samples (n = 349), the total population of AGR-assay-positive samples (n = 643), and the population of ITS-assay-negative, AGR-assay-positive samples (n = 296). Medians are depicted by the horizontal lines, while the box for each plot represents the interquartile range (IQR), and whiskers represent Q3 + (1.5)(IQR) and Q1 – (1.5)(IQR).

Comparing results obtained using the newly described qPCR AGR assay with those generated through testing with a previously described, ribosomal ITS-targeting qPCR assay [20], an analysis of 2,784 human stool DNA extracts was performed. 349 samples were determined to be positive using the ITS-targeting assay, while 643 samples were determined to be positive utilizing the newly described qPCR AGR assay. Of the 349 ITS-assay positives, only two were negative when tested by the new AGR assay. In contrast, of the 643 samples determined to be positive by the AGR assay, 296 were negative when tested by the ribosomal ITS assay (Table 3). This led to a sensitivity of 99.69% for the AGR assay, and an ITS-targeting assay sensitivity of 54.11%. Minimum, maximum, median, and quartile values for the ITS-assay-positive sample population, the AGR-assay-positive sample population, and the AGR-assay-positive, ITS-assay-negative sample population are shown in Fig 3.

**Table 3:** Agreement of assay results upon comparative testing of field-collected stool extracts.

	Ribosomal ITS Assay			
AGR qPCR Assay		Negative	Positive	Total
	Negative	2,139 (76.83%)	2 (0.07%)	2,141 (76.90%)
	Positive	296 (10.63%)	347 (12.47%)	643 (23.10%)
	Total	2,435 (87.46%)	349 (12.54%)	2,784 (100.00%)

**Fig 4:**
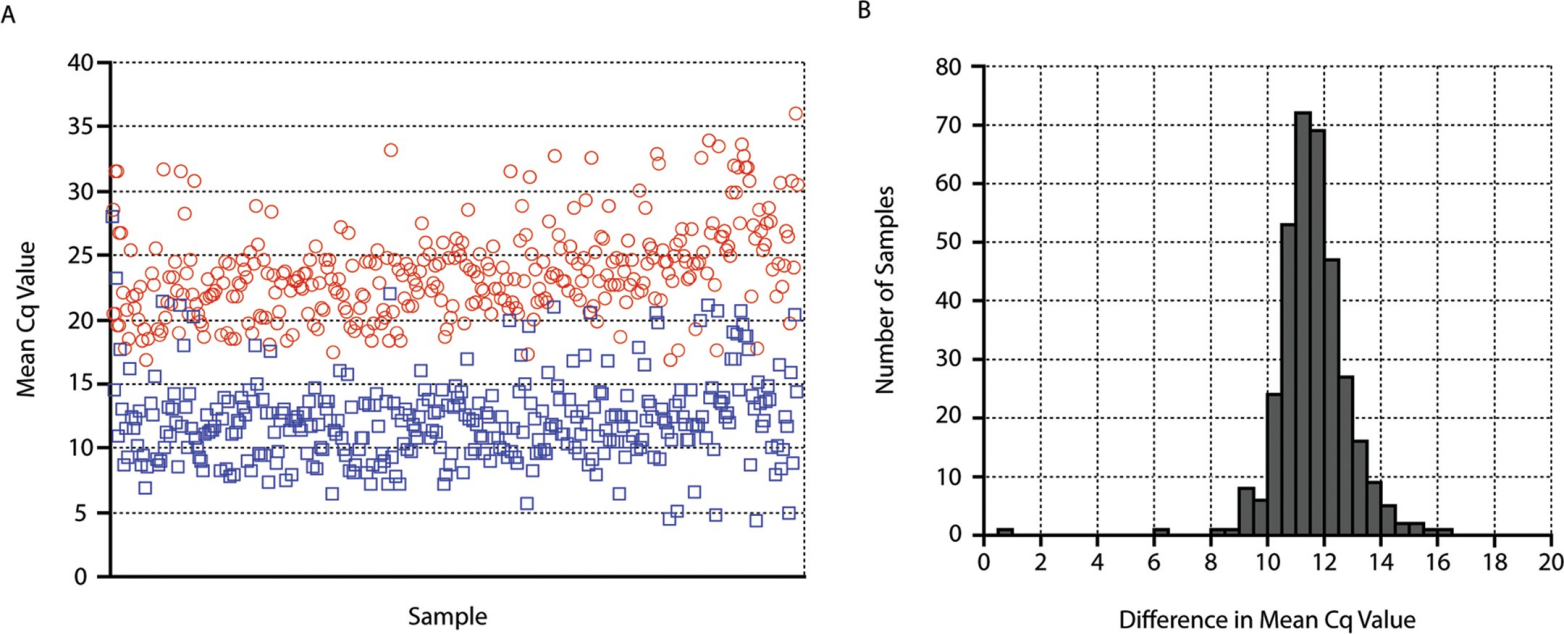
Differences between mean Cq values for all samples co-positive using both the ribosomal ITS-targeting assay and AGR qPCR assay. (A) For each co-positive sample, mean Cq values were plotted for both the ribosomal ITS-targeting assay (red circles) and the AGR assay (blue squares). (B) For each co-positive sample, a difference in mean Cq values was calculated by subtracting the mean value for the ribosomal ITS qPCR assay from the mean value for the AGR qPCR assay. Results were binned by difference and plotted. The average difference across all plotted samples was determined to be 11.51 cycles.

In an attempt to quantify the improvement in reaction sensitivity offered by the new AGR assay, an average reduction in mean Cq value was calculated for all samples which tested positive by both experimental assays, excluding a single sample which produced a lower Cq value when tested using the ribosomal ITS assay (n = 346). To calculate this average reduction in mean Cq, the difference in mean Cq values for each co-positive sample was determined by subtracting the mean Cq value for the ribosomal ITS-targeting assay from the mean Cq value for the AGR assay. The average of these differences was then determined to be 11.51 cycles (range of 0.55–14.99) (Fig 4). This average change in Cq value corresponds to a fold increase in target number between the two qPCR assays of ~3,100, which resulted in the detection of *Ascaris* DNA in nearly twice as many stool samples.

## Discussion

With the expansion of treatment efforts and the resulting declines in infection prevalence and intensity, the sensitivity and specificity of STH diagnostic methods are increasingly important. Post-treatment surveys and population surveillance efforts are only as precise as the tools used to perform them and inconsistent tools may result in mismeasurement or misinterpretation of intervention impact. [31]. As such, diagnostic accuracy is critical for making assessments, and a given study’s programmatic value is inherently tied to diagnostic capability. As OR efforts increasingly work towards the definition of transmission breakpoints, important decisions will be made based upon diagnostically determined prevalence levels under settings of declining parasite burden and decreasing infection intensities. The importance of diagnostic accuracy is embodied by the criteria governing the DeWorm3 cluster randomized trials, which state that “transmission interruption in a cluster will be defined as achieving a prevalence of each STH species of ≤2% …by qPCR 24 months after the final round of MDA” [11]. However, the attainment of a 2% prevalence rate is inherently linked to the test used for prevalence determination. Therefore, understanding diagnostic performance is critical for proper decision making, and maximizing diagnostic sensitivity increases confidence when breakpoint thresholds are attained, minimizing the odds of future recrudescence.

Previously, Easton, et al., described the theoretical limits of detection for both Kato-Katz and PCR as a function of the sample volume used for diagnosis [31]. As such, a 50 mg stool sample, analyzed by PCR has a theoretical limit of detection of 20 eggs per gram, should a single egg be present within the analyzed aliquot. However, Easton and colleagues also point out that with sufficient sensitivity, shed DNA, or DNA resulting from egg degradation could also be detected, allowing for further improvement over microscopy-based techniques that are dependent upon the presence of intact eggs within the sample aliquot tested [31]. While such levels of sensitivity may appear to have reduced importance when one considers that a single adult female *Ascaris* worm has been estimated to shed as many as 200,000 eggs per day [32], egg shedding varies considerably from person to person, and factors such as individual host immunity, geography, age of worm, worm burden, and intervention history can drastically alter patterns of egg production [33]. By selecting a molecular target with dramatically improved copy number, the capacity to detect pathogen signal is greatly improved, theoretically pushing limits of detection to previously impossible levels (Table 4).

**Table 4:** Causes of PCR positivity in the absence of microscopic identification of pathogen.

Circumstance Underlying Microscopic Detection Failure	Mechanism Facilitating qPCR Detection in Absence of Microscopic Detection
• Improper sample storage resulting in degradation of eggs	• Detection of short, tandem DNA repeats does not require intact eggs and repeats remain amplifiable even after significant fragmentation due to tandem arrangement
• Eggs are absent, but gDNA is present	• qPCR will detect target DNA, regardless of the DNA’s source material (eggs, shed tissues, etc.)
• Light infection is missed due to human error	• Molecular detection is not subject to human error or fatigue

Recognizing the need for optimal sensitivity in molecular diagnosis, we previously described the identification of improved qPCR targets for the detection of a number of human-infecting soil transmitted helminths [20–21]. However, due to the unusual phenomenon of chromosome diminution, whereby repeat-enriched portions of the genomic DNA are eliminated between the third and seventh cellular divisions, we were unable to identify an appropriate, novel, high copy-number repeat DNA element within the adult genome of *A*. *lumbricoides*. Recently, due to the publication of a reference-quality germline genome sequence for *A*. *lumbricoides* [26], we have been able to overcome this challenge with the selection of a highly repetitive DNA target that yields vastly superior sensitivity over previously utilized target DNA sequences. Present in both the *A*. *lumbricoides* and *A*. *suum* germline genomes, this target facilitates improved diagnostic detection of all human *Ascaris* infections.

Representing an estimated 8.9% of the germline genome, yet only 120 bp in length, it is not surprising that the DNA target utilized by our new AGR assay facilitated a dramatic decrease in Cq values when compared to qPCR tests based on ribosomal DNA targets. With an estimated genome size of 334 Mb [26], nearly 2.5 x 10^5^ copies of this AGR element are believed to exist per haploid *A*. *lumbricoides* genome. This is in sharp contrast to the estimated 42 copies of ribosomal DNA present in *Ascaris* [34]. Interestingly, assuming similar reaction efficiencies, these copy numbers would suggest a Cq difference of just over 12, in near agreement with the 11.51 mean cycle difference which was determined during the experimental testing of field samples described here. Such drastic improvement in sensitivity should facilitate detection of *Ascaris* DNA at levels well below the quantity which is recoverable from a single egg, a hypothesis further supported by our spiking experiment results (Table 2). The validity of this sensitivity increase was reinforced by the results of the extensive specificity testing which we performed, providing strong evidence that the increased rates of positivity do not result from non-specific, off-target amplification.

It should be noted that a shortcoming of the performed spiking experiment was a failure to utilize an IAC during the DNA extraction procedure. However, as results for spiked sample testing existed for both the AGR assay and the ITS-targeting assay, an assessment of comparative sensitivity remained possible. While it is unfortunate that this failure to include an IAC prevented the drawing of meaningful correlations between Cq values and EPG levels, it is worth mentioning that large OR efforts, such as the DeWorm3 cluster randomized trials, aim to assess transmission break points based solely upon infection prevalence, irrespective of infection intensity [11].

In addition to their direct OR and surveillance functions, sensitive and specific diagnostic tools allow the research community to amass large bodies of accurate data, essential for the expansion and development of novel ideas and methodologies. The increased reliance on such data is seen in the incremental advancement of modeling efforts, a group of tools playing an ever-increasing role in both research and programmatic communities. Similarly, innovative ideas, such as the possibility of utilizing environmental sampling for STH surveillance [35] have historically been hampered by insufficient diagnostic options. However, with a resurging interest in these alternative methodologies [36–37], the availability of more sensitive tools will be critical, as such samples will likely rely upon larger sample masses, resulting in the dilution of molecular signal. Furthermore, while it is likely that future technologies will eventually render the current methods of qPCR-based diagnostics obsolete, prevalent targets will remain prevalent and may prove useful as new technologies come online. As such, the discovery of optimal targets should have a lasting impact on the field of infectious disease diagnostics.

While detection of parasite DNA target at sub-single egg concentrations greatly improves the sensitivity of detection, expanded sensitivity can also result in a potential complication. The issue is that higher copy-number DNA targets, coupled with excellent qPCR efficiencies, render assays increasingly susceptible to the possibility of sample-to-sample contamination. Such concerns are especially valid when the transfer of the technology to endemic countries is a priority. Deployment of such assays to varied laboratory environments can lead to an increased risk of false positive and false negative results. Accordingly, highly sensitive qPCR assays require added attention to detail, and highlight the need for a renewed programmatic focus on proper training and project oversight. Equally important, appropriate quality assurance and quality control practices must be implemented, as must the use of consistent and standardized procedures and controls. Recognizing this need, options for external laboratory quality assessment are growing, and participation in assessment programs such as the Helminth External Molecular Quality Assessment Scheme offered by the Dutch Foundation for Quality Assessment in Medical Laboratories (SKML) should be considered whenever possible. Submitting to such external evaluations will help to ensure the accuracy of results and the inter-lab comparability of data.

Although infrequently voiced, an additional concern stems from the sometimes stated belief that optimization of a diagnostic assay can theoretically lead to the development of a test that is “too sensitive”. The argument has been made that the detection of sub-cellular levels of DNA from cellular debris may result in the false attribution of a “positive” status to individuals who are not actually harboring active infection [38]. Similarly, for certain pathogens, detection of an individual microorganism may lead to diagnostic “positivity” under non-pathogenic concentrations [39–40]. However, such concerns are more relevant in the context of the clinical diagnosis of an individual patient. It is certainly true that sub-infectious levels of a pathogen may not pose a significant risk to the individual patient. Yet when used in a surveillance capacity, even sub-clinical levels of pathogen, or pathogen-derived material, are indicative of pathogen presence within the population. Oftentimes, such sub-clinical levels of pathogen may still pose a transmission risk within the community, facilitating persistence or providing an early indication of possible infection recrudescence [41–42]. As such, when used for surveillance purposes, maximizing sensitivity should always be the diagnostic goal. However, it is equally important to remember that presence of pathogen signal is not necessarily an indicator of the potential for transmission. Factors such as single sex infections and expulsion of pathogen material can result in sample positivity despite failing to pose a transmission risk. For this reason it is critical that assay results be interpreted in the context of the study environment. Should the aims of a particular study dictate that only more heavily infected samples be of interest, a Cq value cutoff could be imposed, allowing the investigators to effectively filter out “light”, potentially sub single-egg positive results without requiring changes to the testing procedure.

By targeting a highly repetitive element of the germline genome, the AGR qPCR assay described here has the capacity to greatly improve the sensitivity of detection of human *Ascaris* infections. This improvement should aid both operational research and programmatic efforts, increasing the accuracy of diagnostic results and facilitating better-informed decision making processes. Given the vast global prevalence of human *Ascaris* infection, the addition of this novel assay to the list of available molecular tools is of considerable significance.

## Supporting information

S1 Checklist STARD checklist. Locations within the manuscript addressing each checklist item are indicated. This checklist is intended to provide the reader with criteria for assessing potential study biases and to consider the potential for generalizability of the results reported.
(PDF)

S1 Fig Amplification plot for qPCR reactions used to calculate reaction efficiency. Through replicate testing of titrated plasmid DNA containing a single copy of the reaction target sequence, amplification curves were used to determine mean Cq values for reactions occurring with each concentration of template.
(TIF)
