# Supplementary figures and images for: Targeting a highly repeated germline DNA sequence for improved real-time PCR-based detection of Ascaris infection in human stool
Source: PLoS Negl Trop Dis. 2019 Jul 22;13(7):e0007593. doi: 10.1371/journal.pntd.0007593 (PMC6675119; doi:10.1371/journal.pntd.0007593)

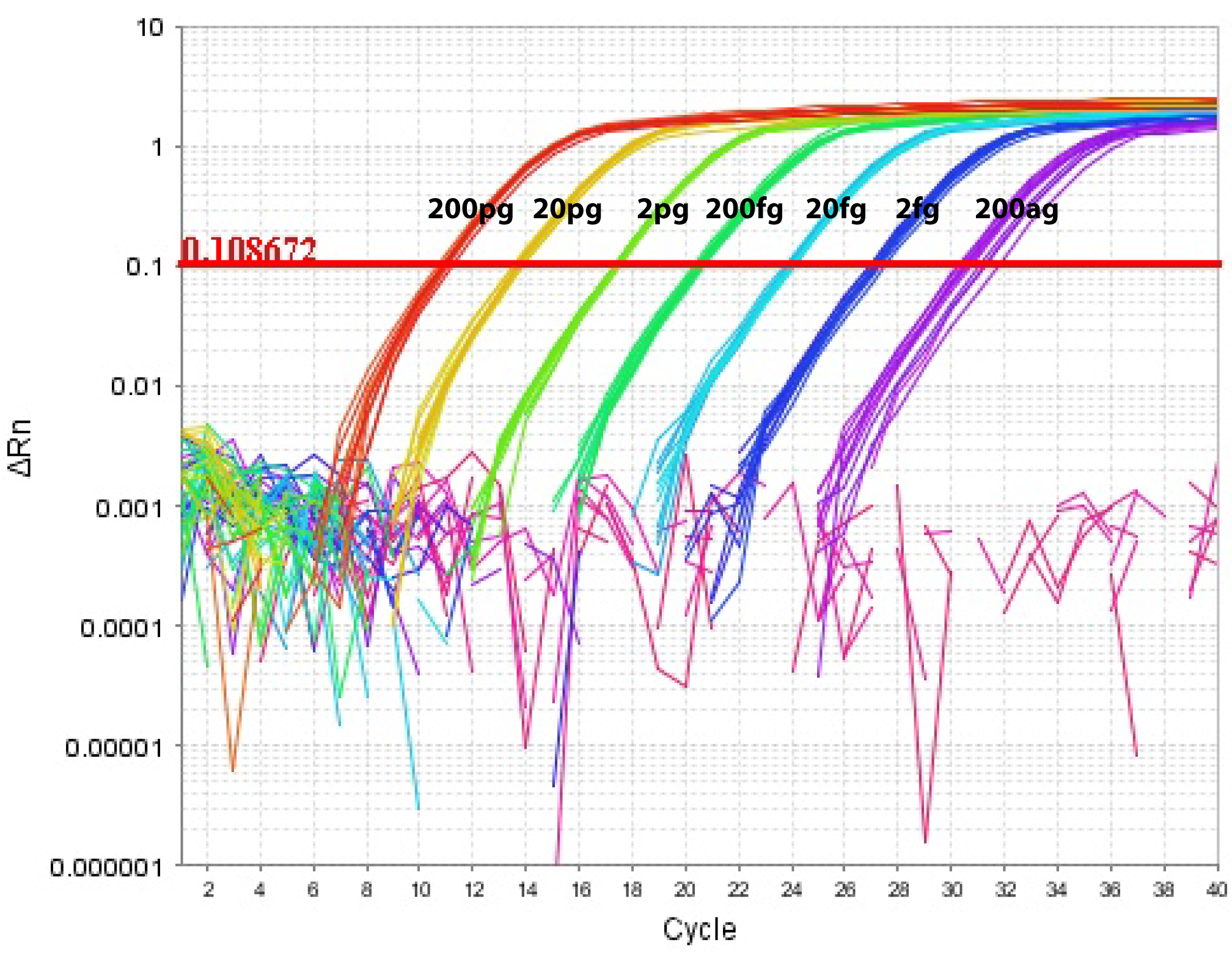

Supplement: S1 Fig — Through replicate testing of titrated plasmid DNA containing a single copy of the reaction target sequence, amplification curves were used to determine mean Cq values for reactions occurring with each concentration of template. (TIF) [file pntd.0007593.s002.tif]
